# Phosphor Ceramic Composite for Tunable Warm White Light

**DOI:** 10.3390/ma17133187

**Published:** 2024-06-29

**Authors:** Ross A. Osborne, Nerine J. Cherepy, Peter S. Bleier, Romain M. Gaume, Stephen A. Payne

**Affiliations:** 1 Lawrence Livermore National Laboratory, 7000 East Ave, Livermore, CA 94550, USA; 2 Department of Material Science and Engineering, University of Central Florida, 12760 Pegasus Dr, Orlando, FL 32816, USA

**Keywords:** phosphors, composites, ceramics, lighting, LEDs

## Abstract

Composite phosphor ceramics for warm white LED lighting were fabricated with K_2_SiF_6_:Mn^4+^ (KSF) as both a narrowband red phosphor and a translucent matrix in which yellow-emitting Y_3_Al_5_O_12_:Ce^3+^ (YAG) particles were dispersed. The emission spectra of these composites under blue LED excitation were studied as a function of YAG loading and thickness. Warm white light with a color temperature of 2716 K, a high CRI of 92.6, and an R9 of 77.6 was achieved. A modest improvement in the thermal conductivity of the KSF ceramic of up to 9% was observed with the addition of YAG particles. In addition, a simple model was developed for predicting the emission spectra based on several parameters of the composite ceramics and validated with the experimental results. The emission spectrum can be tuned by varying the dopant concentrations, thickness, YAG loading, and YAG particle size. This work demonstrates the utility of KSF/YAG composite phosphor ceramics as a means of producing warm white light, which are potentially suitable for higher-drive applications due to their increased thermal conductivity and reduced droop compared with silicone-dispersed phosphor powders.

## 1. Introduction

Light-emitting diodes (LEDs) are rapidly replacing older lighting technology due to their improved efficiency, lifetime, and color quality, reduced rare earth element usage, decreased cost, and lack of toxic materials. Phosphor-converted white LEDs (pc-LEDs) are typically used for illumination, in which an efficient blue LED is combined with one or more phosphors to down-convert some of the blue light to other wavelengths and produce white light. Y_3_Al_5_O_12_:Ce^3+^ (YAG) is widely used as a yellow phosphor, which used alone with a blue LED can produce efficient cool white light [1]. The emission maximum of YAG can be shifted by substitution with various ions (e.g., Lu, Gd) [2] or the addition of other emitters (e.g., Mn). However, to produce efficient warm white light, which is generally preferred in many settings, with a good R9 (a metric for the red content of the light) and a high color rendering index (CRI), an additional red phosphor is required [3].

The most widely used red phosphors are CaAlSiN_3_:Eu^2+^ (CASN), Sr_2_Si_5_N_8_:Eu^2+^, and K_2_SiF_6_:Mn^4+^ (KSF) [4,5]. The Eu^2+^-doped nitride phosphors suffer from a broad emission spectrum extending beyond 650 nm, where the human eye is insensitive, which degrades the luminous efficacy of the LED lamp [3,6]. KSF is a particularly useful red phosphor due to its narrow emission around 630 nm, as well as its strong absorption at 450 nm and high quantum efficiency [5,7].

Phosphor powders are typically dispersed in a silicone matrix that is placed on top of the LED, known as phosphor in silicone (PiS); however, the silicone matrix has a low thermal conductivity (0.18 W/m-K) [8], reducing heat flow out of the phosphors and limiting the light flux that can be used to prevent overheating of the phosphors. It can also be subject to discoloration and degradation over time [9]. Phosphor in glass (PiG) is an approach that has been explored to address these issues, as it has higher thermal conductivity (up to 2.32 W/m-K) and better environmental stability [8,10,11]. However, only glasses with a low glass transition or melting temperature are suitable for use with KSF due to its low decomposition temperature (<600 °C), unlike YAG, ruling out high-thermal-conductivity silica glasses requiring a sintering temperature of 1250 °C [10,11]. KSF-containing PiG deposited on a sapphire substrate has been reported [12], as well as LuAG:Ce-containing PiG coated with a KSF film to produce white light [13]. PiG with other phosphor mixtures has also been utilized to produce white light [14,15]. Phosphor ceramics or single crystals, such as YAG, have also been used, especially for high-light-flux applications like blue laser-based lighting, due to their higher thermal conductivity, low scatter, and environmental stability [9]. However, YAG ceramics can be difficult to produce, generally requiring the use of nanopowders, high processing temperatures (~1780 °C), and multiple steps [16]. CaAlSiN_3_:Eu^2+^ red phosphor ceramics offering improved thermal conductivity and stability have been demonstrated, but with broad red emission and the requirement for high processing temperatures [17,18,19]. KSF single crystals 1 mm in size have been grown and exhibited improvements over powdered KSF in thermal quenching, heat dissipation, and moisture stability [20].

We previously reported on the development of a KSF phosphor ceramic that exhibited improved droop behavior and thermal conductivity compared with phosphor in silicone [21,22]. Additionally, these phosphor ceramics can be fabricated at relatively low temperatures (≤400 °C) in a single hot-pressing step, unlike YAG ceramics. In this paper, we hypothesize that composite phosphor ceramics consisting of YAG particles embedded in a KSF matrix would be a viable method for producing warm white light with desirable characteristics, such as a high color rendering index and R9 for phosphor-converted LEDs. This would allow us to take advantage of the improved thermal conductivity and droop behavior of the KSF phosphor ceramic while also producing warm white light. In addition, YAG particles introduce optical scatter into KSF ceramics. The presence of a controlled amount of optical scatter has been shown to be beneficial in reducing totally internally reflected (TIR) light and thus improve color homogeneity with respect to the viewing angle of the light, as well as efficiency [9,23]. Finally, bulk KSF phosphors are more resistant to degradation by water than KSF powders [20]. Here, we focus on the effect of the sample thickness and YAG loading fraction on the emission spectra. Other parameters, such as the doping concentrations of Mn and Ce and the YAG particle size, were held constant.

Additionally, an optical model was developed to predict the emission spectrum of these composite ceramics based on their properties, such as thickness, YAG loading fraction, YAG particle size, and dopant concentrations. This approach could be applied to other composite phosphor ceramics or phosphor in glass to aid the fabrication process. To the authors’ knowledge, this is the first example of a composite phosphor ceramic for LED lighting in which both the ceramic matrix and the embedded particles are active phosphors.

## 2. Materials and Methods

**Figure 1:**
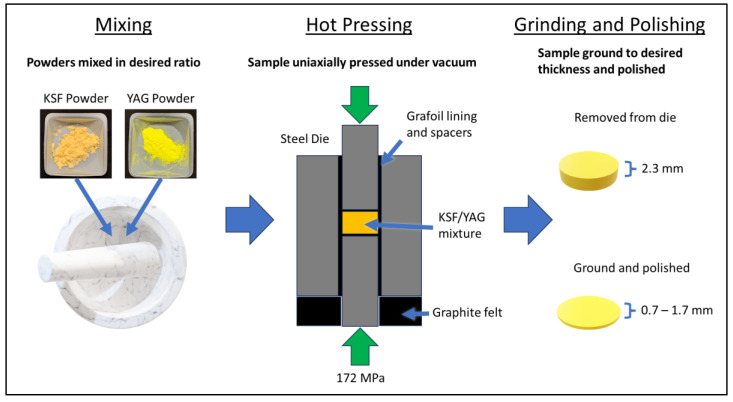
Synthesis process for composite phosphor ceramics.

The synthesis procedure for the phosphor ceramics is illustrated in Figure 1. Phosphor powders of K_2_SiF_6_:Mn^4+^ (KSF, Current Chemicals, Cleveland, OH, USA) and Y_3_Al_5_O_12_:Ce^3+^ (YAG, Intematix, Fremont, CA, USA) were weighed to the desired ratios (1 g in total) and hand-mixed in a mortar without grinding. Samples were produced with YAG loading fractions of 0, 2.5, 4, 5, and 7.5 wt%. Above 7.5 wt% YAG, the samples became too highly scattered to allow sufficient blue light transmission at the thicknesses studied. The concentrations of the dopants Mn and Ce were analyzed by Galbraith Laboratories, Inc. (Knoxville, TN, USA) via inductively coupled plasma optical emission spectroscopy (ICP-OES) and found to be 0.048 wt% and 0.75 wt%, respectively. The phosphor powders were handled in an argon-filled glovebox to prevent the adsorption of water. The mixed powders were loaded into a 12.7 mm diameter steel die lined with pyrolytic graphite foil, which helped prevent the cracking of the sample. The die body was supported by a ring of compressible graphite felt to allow for a more symmetrical densification (also known as dual-action pressing). The die was then loaded into a uniaxial hot press, pressed under vacuum (≤3.0 × 10^−3^ mTorr) at approximately 172 MPa (25,000 psi) and 300–400 °C, and held for 1 h. The optimal pressing conditions were experimentally determined by varying the temperature and pressure. Above ~400 °C, the samples darkened, and below ~300 °C, the translucency was reduced. The maximum working pressure for the die/hot press was used, as lower pressure resulted in less translucency. The temperature ramp rate was 10 °C/min (upward and downward), and pressure was not applied until the dwell temperature was reached. The pressure was released at the end of the dwelling. The surfaces of the ceramics were polished with a solution of sodium lauryl sulfate, water, and 0.5 μm diamond grit on a soft pad. The densities of the samples were measured using the Archimedes method with water.

The emission spectra of the ceramic samples were measured at various thicknesses. Samples were first measured at 1.7 mm thickness; they were then ground and polished to 1.0 mm, and finally to 0.7 mm, so that, assuming homogeneity, all the change in the emission spectrum with respect to thickness for a particular YAG loading fraction is due to the change in thickness rather than sample-to-sample variation. The optical absorption spectra of the ceramics were measured using a Thermo Evolution 220 UV-Vis spectrometer. The blue-LED-excited spectra of the ceramics were measured by placing a blue LED (λ_max_ = 450 nm) behind the ceramics at the input slit of a Princeton Instruments/Acton Spec 10 spectrograph coupled to a thermoelectrically cooled CCD camera.

Scanning electron microscopy (SEM) and energy dispersive spectroscopy (EDS) were performed using a Zeiss FEI SEM with an accelerating voltage of 15 keV. The samples were coated with a 5 nm thick carbon layer to provide electrical conductivity.

X-ray diffraction (XRD) was performed on the ceramic samples with a Bruker AXS D8 ADVANCE X-ray diffractometer with a Cu anode (Kα1 = 0.1540598 nm).

Thermal conductivity measurements were performed via the Transient Plane Source method (TPS) using a Hotdisk AB TPS 2200 with a 2 mm radius Kapton sensor. Stainless steel verification samples were used to guarantee the accuracy of the TPS and sensors, and the protocol for a dense ceramic with low porosity was conducted. The heating power was 400 mW with a measurement time of 10 s, recorded between 50 and 150 °C in 25 °C increments with a probing depth of 3.5 mm.

## 3. Results and Discussion

**Figure 2:**
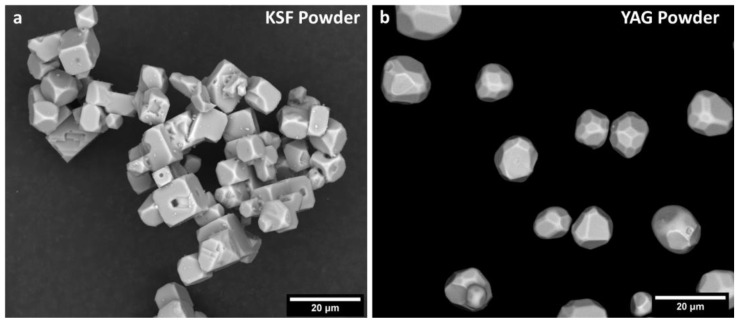
(**a**) KSF powder and (**b**) YAG powder used to make the phosphor ceramics.

The starting powders of YAG and KSF, imaged via SEM, are shown in Figure 2. The KSF particles are cuboidal and agglomerated, while the YAG particles are roughly spherical and unagglomerated. The average particle sizes of the powders were 10 μm and 13.6 μm for KSF and YAG, respectively, as indicated by the manufacturers and verified via microscopy.

**Figure 3:**
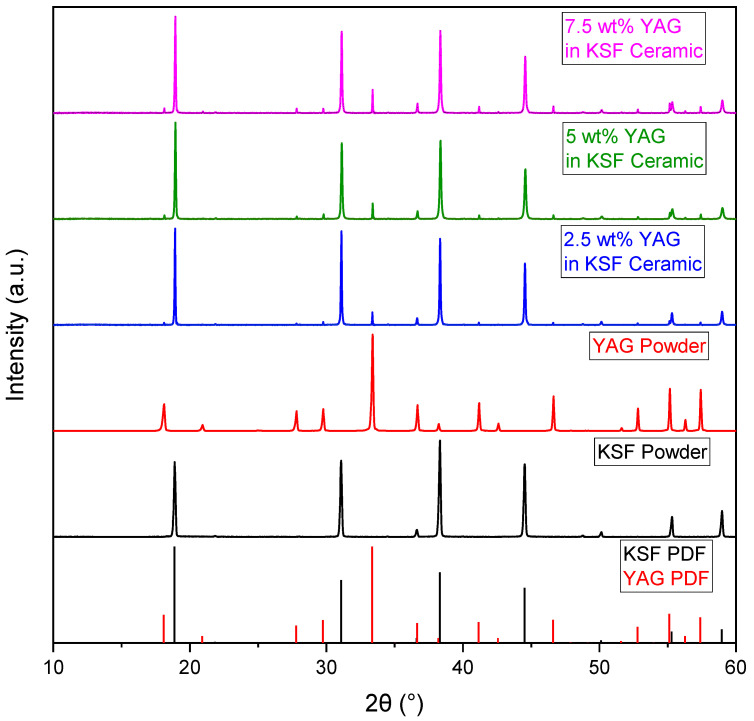
XRD patterns of the raw powders and ceramic samples with 2.5, 5, and 7.5 wt% YAG loading fractions, along with the powder diffraction reference patterns for the KSF (PDF file 04-006-8962) and YAG (PDF file 04-007-2667) crystal phases.

The XRD patterns of the raw phosphor powders and ceramics with various loading fractions of YAG are shown in Figure 3. The raw powders are pure-phase KSF and YAG. As expected, the only phases identified in the ceramics are the cubic KSF and YAG phases, indicating that the hot-pressing conditions do not induce reactions between the constituents and form undesired phases, which would degrade the quantum efficiency or translucency. This is unsurprising given that the hot-pressing temperature is far below the melting point of YAG (1970 °C). Furthermore, there are no reactions with the environmental atmosphere, the steel die, or the graphite foil. The intensities of the peaks also match well with the theoretical values, indicating that there is no preferential grain orientation of KSF or YAG. The densities of the 0, 2.5, 4, 5, and 7.5 wt% YAG samples were measured at 2.70, 2.72, 2.73, 2.73, and 2.76 g/cm^3^, respectively. Since YAG has a higher density than the pure-KSF ceramic, an increasing trend in density with the YAG loading fraction is expected. The theoretical densities of the samples based on the volume fractions of YAG and KSF, using 4.560 g/cm^3^ and 2.700 g/cm^3^ for pure YAG and KSF, respectively, are 2.700, 2.728, 2.745, 2.756, and 2.785 g/cm^3^ (in the same order). The measured densities of the composite ceramics are less than 1% lower than the theoretical densities. This small discrepancy may be due to the accuracy of the measurement, or possibly to the YAG particles introducing a small amount of porosity, which lowers the density slightly.

**Figure 4:**
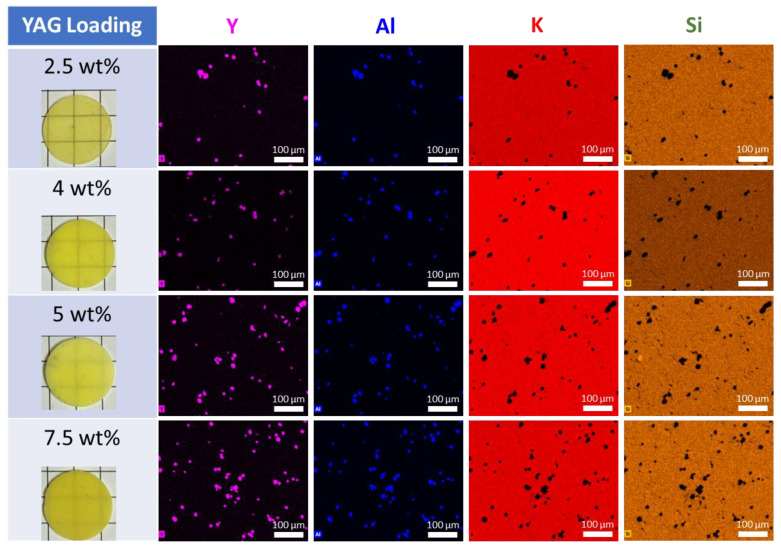
Energy-dispersive X-ray spectroscopy maps of the polished surfaces of samples with 2.5, 4, 5, and 7.5 wt% YAG loading fractions (pictures of samples at left).

**Figure 5:**
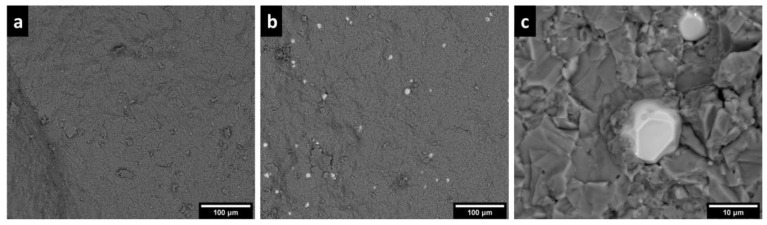
Backscatter electron micrographs of a fracture surface of (**a**) KSF-only ceramic and (**b**) composite ceramic, and (**c**) close-up of YAG particles in a composite ceramic.

Figure 4 shows energy-dispersive X-ray spectroscopy (EDS) maps of constituent elements in the samples. The YAG particles appear as hotspots on the Y and Al maps and as dark spots on the K and Si maps. The YAG particles are well dispersed, which is important for homogeneous absorption and emission throughout the ceramics. The YAG particles have clearly defined boundaries, indicating the lack of reaction or diffusion of ions from YAG to KSF or vice versa. SEM imaging (Figure 5) shows that the morphology of the KSF matrix does not change upon the addition of YAG particles and that the KSF grains deform around the YAG particles, which maintain their original morphology.

**Figure 6:**
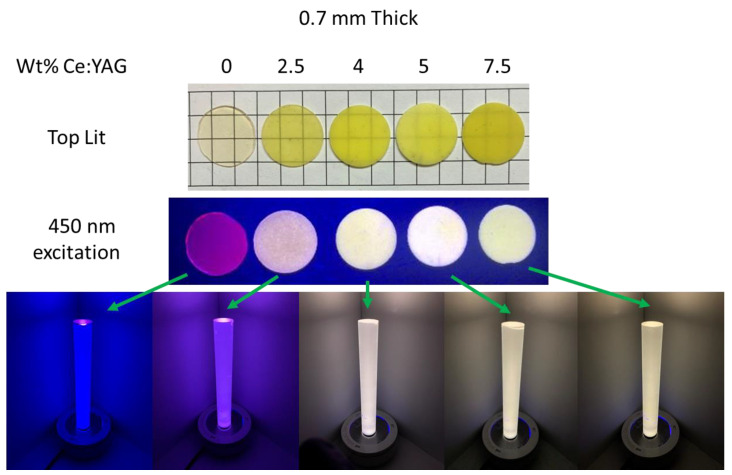
(**Top**) Pictures of the samples under room lights and 450 nm excitation. (**Bottom**) A diffuse light rod sitting on top of the samples irradiated from beneath with a blue LED.

Photographs of the samples are shown in Figure 6. The translucency of the samples decreases with an increasing YAG loading fraction due to increased scatter. The samples were excited on one side with a blue LED, and a diffuse light rod was placed on top to view the emitted light (bottom of Figure 6). The light color scattering out of the rod changes as a function of the YAG loading fraction and sample thickness. The images in Figure 6 were all taken at a sample thickness of 0.7 mm. The light is blue for the sample without YAG, since most of the LED light is transmitted due to the low Mn concentration and thin sample. At 2.5 wt% YAG, the light becomes purple due to the yellow light contributed by the YAG particles and a longer path length for the blue light, caused by increased scatter, leading to less transmitted blue light. At 4 wt% YAG, white light is achieved, and at 5 and 7.5 wt% YAG, the white light becomes warmer.

**Figure 7:**
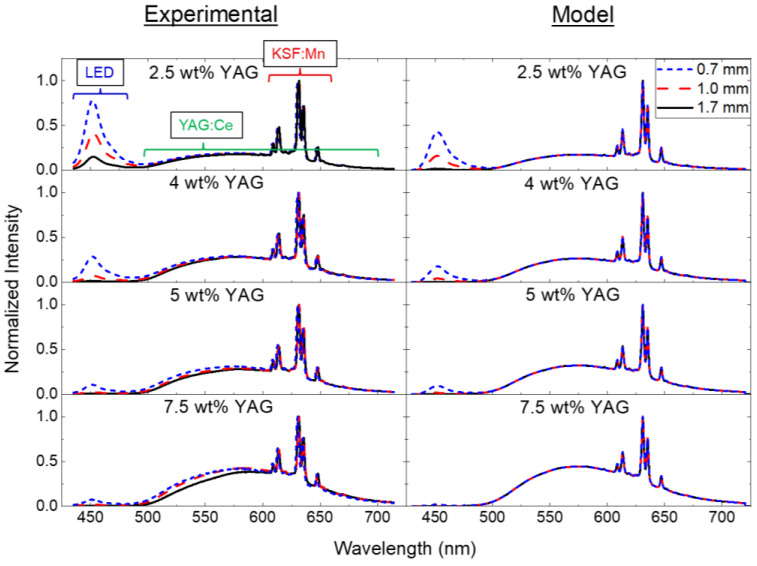
Experimental and modeled emission spectra of the samples excited by a blue LED with various YAG loading fractions at different thicknesses. The spectral contributions attributed to the LED, YAG:Ce, and KSF:Mn are labeled in the top left panel.

The emission spectra of the samples are shown in Figure 7. The spectra are comprised of a combination of transmitted blue LED light (centered at 450 nm), yellow YAG:Ce emission (broadly ranging from ~500 to 700 nm), and red KSF:Mn emission (sharp peaks in the range of 600–650 nm). All spectra are normalized to the maximum of the KSF emission peak (at 631 nm). The YAG-to-KSF emission ratio increases as a function of increasing YAG loading, as expected, while the blue LED light is inversely related to the YAG loading and sample thickness. The spectra predicted by the optical model, described below, are also shown and are qualitatively in close agreement with the experimental data.

A simplified model was developed to predict the emission spectra of composite ceramics with various YAG loading fractions and thicknesses, in the same geometry as that in which the emission spectra were measured (light emitted from the face opposite the LED). To simulate the spectrum, *S_total_*, arising from the KSF/YAG composite irradiated by a blue LED, we need to evaluate the contributions of each individual spectra (*S_i_*, with linear coefficients of *a_i_*):

(1) $$S_{total}=a_{red}S_{KSF}\left(\lambda \right)+a_{green}S_{YAG}\left(\lambda \right)+a_{blue}S_{diode}\left(\lambda \right)$$

By taking scattering and absorption into account for the “first” encounter of the blue light with the composite, and by referring to the quantities defined in Table 1, we use

(2) $$a_{blue}=a_{0}\exp\;\left[-\left(\alpha_{KSF}\left(1-f_{vol}^{YAG}\right)+\alpha_{YAG}f_{vol}^{YAG}\right)l\right)exp\;(-\beta_{scat}l)]$$

where *l* is the sample thickness, $f_{vol}^{YAG}$ is the volume fraction of YAG particles (Equation (3)), α_YAG_ is the YAG absorption coefficient, α_KSF_ is the KSF absorption coefficient, and β_scat_ is the scattering coefficient. The absorption coefficients were calculated by multiplying the Ce or Mn absorption cross-section by the Ce or Mn number density (*N_Ce_* and *N_Mn_*), respectively (Equations (4) and (5)).

(3) $$f_{vol}^{YAG}=\frac{f_{wt}^{YAG}}{\rho_{YAG}}÷\left(\frac{f_{wt}^{YAG}}{\rho_{YAG}}+\frac{1-f_{wt}^{YAG}}{\rho_{KSF}}\right)$$

(4) $$\alpha_{YAG}=\sigma_{Ce}N_{Ce},\;\mathrm{where}\;N_{Ce}=f_{wt}^{Ce}\left(\frac{\rho_{YAG}}{m_{Ce}}\right)N_{A}$$

(5) $$\alpha_{KSF}=\sigma_{Mn}N_{Mn},\;\mathrm{where}\;N_{Mn}=f_{wt}^{Mn}\left(\frac{\rho_{KSF}}{m_{Mn}}\right)N_{A}$$

where $f_{wt}^{Ce}$ and $f_{wt}^{Mn}$ are the weight fractions of Ce and Mn in YAG and KSF, respectively. Since the YAG particles are large compared with the wavelength of light, the scattering coefficient is calculated geometrically in terms of the reflections off of the YAG particles:

(6) $$\beta_{scat}=f_{vol}^{YAG}\frac{3}{2d_{YAG}}$$

where *d_YAG_* is the average YAG particle diameter, measured at 13.6 μm. In this case, all the scattered blue light is presumed to remain in the composite.

Next, we calculate the green emission from YAG with the following:

(7) $$a_{green}=a_{0}\left(\frac{\alpha_{YAG}f_{vol}^{YAG}}{\alpha_{KSF}\left(1-f_{vol}^{YAG}\right)+\alpha_{YAG}f_{vol}^{YAG}}\right)[1-exp\;(-\left(\alpha_{KSF}\left(1-f_{vol}^{YAG}\right)+\alpha_{YAG}f_{vol}^{YAG}\right)l)]$$

where the first term separates out the fraction of light absorbed by YAG and KSF. Similarly, for the red light, we have the following:

(8) $$a_{red}=a_{0}\left(\frac{\alpha_{KSF}(1-f_{vol}^{YAG})}{\alpha_{KSF}\left(1-f_{vol}^{YAG}\right)+\alpha_{YAG}f_{vol}^{YAG}}\right)[1-exp\;(-\left(\alpha_{KSF}\left(1-f_{vol}^{YAG}\right)+\alpha_{YAG}f_{vol}^{YAG}\right)l)]$$

This represents the first of potentially many interactions with the blue light, as these same equations are employed a second time for *a*_1_, where

(9) $$a_{1}=1-{\left(a_{blue}+a_{green}+a_{red}\right)}_{0}$$

defining *a*_0_ = 1. This process is repeated *i* times for each subsequent *a_i_* by subtracting the sum of all the blue light absorbed or transmitted in previous interactions until convergence:

(10) $$a_{i+1}=1-\Sigma_{1}^{i}{\left(a_{blue}+a_{green}+a_{red}\right)}_{i}$$

Finally, we add up all the individual blue, green, and red contributions to generate the fitted spectrum of the composite. To fit the data, the known values of experimental variables were used in the model calculations. Given the complexity of modeling the KSF and YAG absorptions in the presence of multiple scatter events (up to 20), the impact of total internal reflection, and the distribution of particle sizes, we conclude that our simplified theory adequately accounts for the basic physics that is operative in the composite samples.

The coefficient of determination (R-squared) for each model prediction with respect to the experimental data was calculated for each sample condition, and the results are presented in Table 2. These data demonstrate that the model agrees with the experimental data quite well, with an R-squared of up to 0.97. The worst fits for the model are those with the lowest YAG loading fraction. This is because the model predicts less blue light transmission than that measured in the experimental data. This discrepancy at low YAG loading may be due to blue light escaping out the sides of the samples via total internal reflection, which was not accounted for in the model. Additionally, the YAG-to-KSF emission ratio is unaffected by the sample thickness at each respective YAG loading fraction in the model, since the YAG loading fraction does not change with the sample thickness. However, in the experimental data, there is a slight decrease in the YAG emission at greater sample thicknesses, particularly for the 5 and 7.5 wt% samples (Figure 7). This may be due to parasitic absorptive defects in the ceramic, which are not considered in the model.

This model can be applied to guide the fabrication of composite phosphor ceramics to produce a desired emission spectrum. The parameters affecting the emission spectrum can be manipulated; these include the Ce and Mn doping concentrations, the YAG particle size and loading fraction, the thickness, and the individual garnet and cubic fluoride emission spectra, which can be tuned through elemental substitution. This approach is applicable to other composite phosphor ceramics or phosphor in glass simply by substituting the spectra and parameters of the phosphors being used.

**Figure 8:**
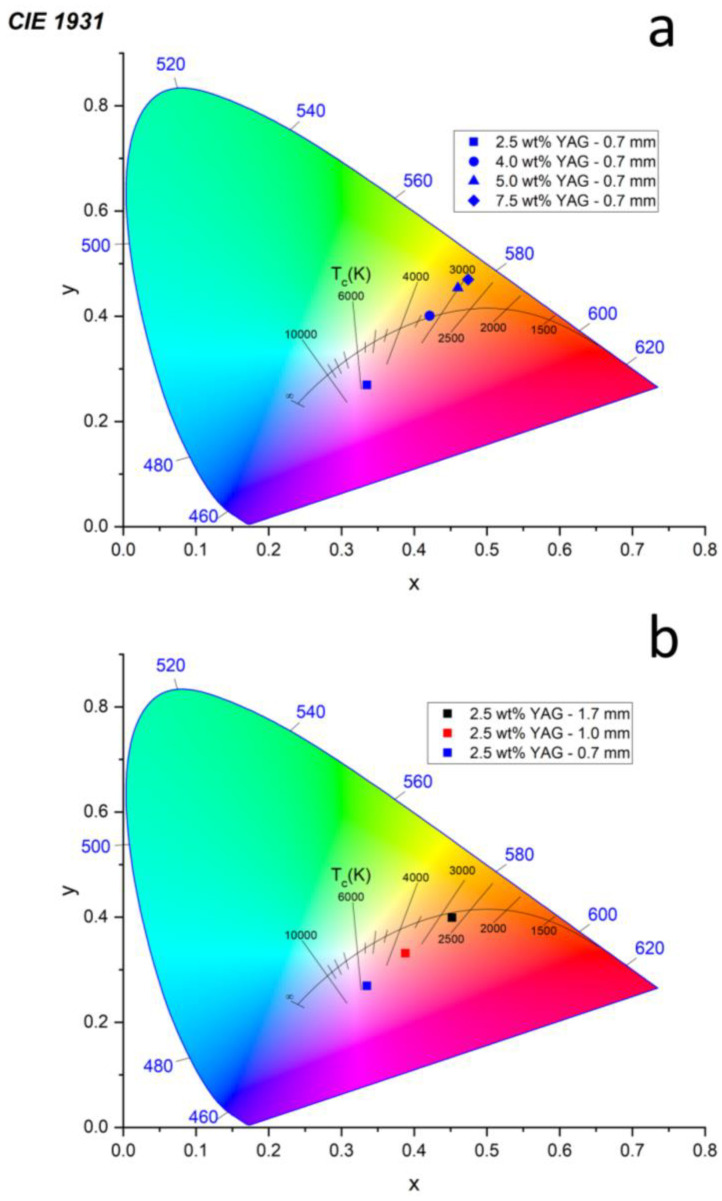
CIE 1931 color coordinate plot of the samples along with the blackbody radiation curve as a function of their (**a**) YAG loading (thickness held constant at 0.7 mm) and (**b**) thickness (YAG loading held constant at 2.5 wt%).

**Figure 9:**
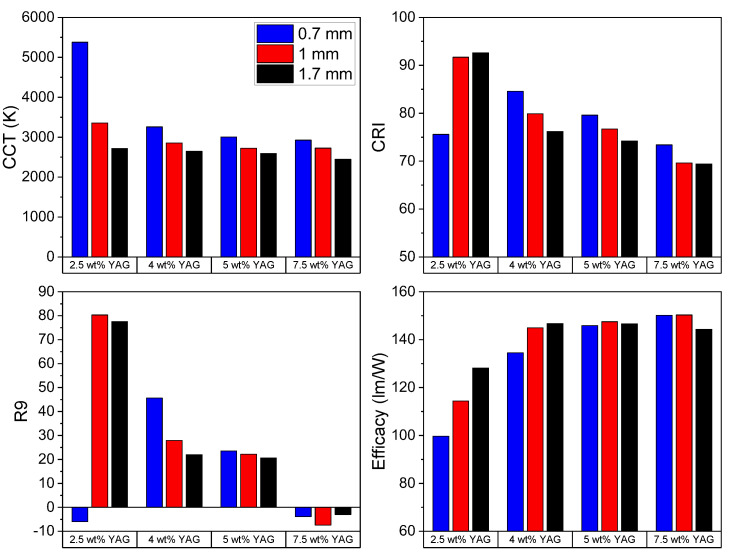
Calculated correlated color temperature (CCT), color rendering index (CRI), R9, and efficacy of the samples.

Two subsets of samples are plotted on CIE color coordinate graphs in Figure 8, and the values of the correlated color temperature (CCT), the color rendering index (CRI), R9, and the estimated efficacy of all samples, obtained using the spreadsheet developed by Li and Luo [24], are shown in Figure 9. The color temperature decreases from about 6000 K to 3000 K with an increase in the YAG loading fraction from 2.5 to 7.5 wt% (Figure 8a) due to decreased blue light transmission and greater YAG emission. Likewise, the color temperature also decreases with increasing sample thickness (Figure 8b) due to decreased blue light transmission. Two samples are close to the blackbody radiation curve: 2.5 wt% YAG—1.7 mm (CCT = 2716 K, x = 0.452, y = 0.399) and 4 wt% YAG—0.7 mm (CCT = 3258 K, x = 0.421, y = 0.401). For reference, the CIE coordinates for a standard tungsten filament bulb with a CCT of 2856 K are x = 0.4476 and y = 0.4074 [25], which are close to the values for the aforementioned samples. All other samples either have too much or too little blue light transmission to fit directly on the blackbody curve. Several samples have desirable CRI (>80) and R9 (>20) values. The estimated efficacy tends to increase with the YAG loading fraction due to human eye sensitivity peaking in the green. Table 3 compiles the CCT, CRI, R9, estimated efficacy, and CIE color coordinates of all the samples.

**Figure 10:**
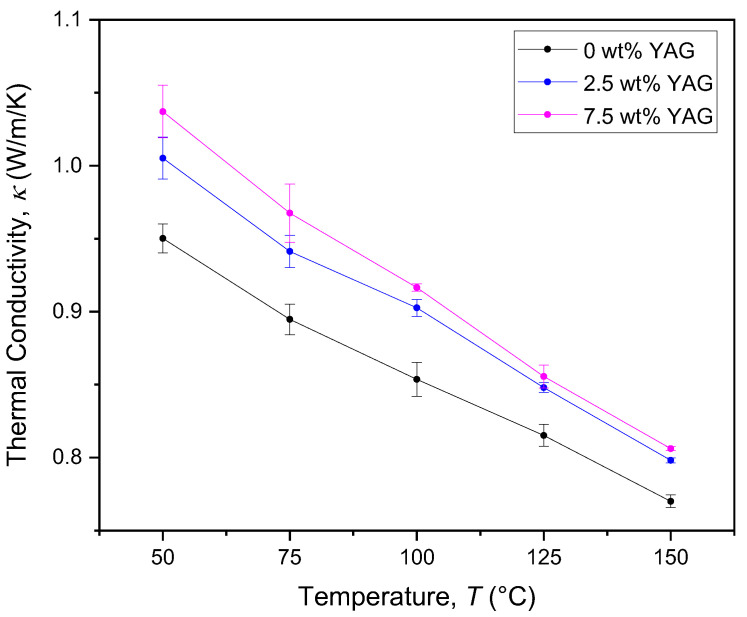
Thermal conductivity as a function of temperature for various loading fractions of YAG in a Mn:KSF matrix.

The thermal diffusivity of the composites was measured as a function of temperature for various loading fractions of YAG (Figure 10). As expected, the thermal conductivity increases alongside an increase in constituent YAG [26], with the greatest impact (a 9% increase for 7.5 wt% YAG) seen at around 50 °C. At higher temperatures, increased constituent YAG in the composition has a diminishing effect due to solute-atom scattering being less impactful in comparison to intrinsic phonon scattering, which scales with temperature [27]. The thermal conductivity of the pure KSF decreases to 0.77 W/m-K at 150 °C, in contrast to our previously reported value of 1.05 W/m-K at 200 °C [21]. This new value is believed to be more accurate since the thermal conductivity is expected to be inversely correlated with temperature in an insulating material.

## 4. Conclusions

Composite phosphor ceramics producing warm white light were fabricated by employing KSF as an active narrow red phosphor matrix containing dispersed yellow-emitting YAG particles. There was no evidence of a reaction between these two materials during the low-temperature hot-pressing step, in which the KSF was deformed around the YAG particles to form a translucent solid ceramic. The emission spectra of these ceramic composites under blue LED excitation were studied as a function of YAG loading from 2.5 to 7.5 wt% and thickness from 1.7 mm to 0.7 mm. Warm white light with desirable emission characteristics, such as a color temperature of 2716 K, a high CRI of 92.6, and an R9 of 77.6, was achieved. The color temperature varied from 2450 to 5380 K within the parameter space studied. A modest increase of up to 9% in thermal conductivity was seen with the addition of YAG particles, with a maximum of 1.04 W/m-K for 7.5 wt% YAG. These results validate the feasibility of this material for producing warm white light.

To help understand these composites and guide their fabrication, a simple model was developed for predicting the emission spectra based on several parameters of the composite ceramics. The model was found to be in good agreement with the experimental results, validating its usefulness. This model could be easily adapted to other composite phosphor ceramics or PiG, where it may help guide fabrication for desired emission spectra. The emission spectra of KSF/YAG composite phosphor ceramics can be tuned through the dopant concentrations, thickness, YAG loading, YAG particle size, and ionic substitutions of KSF/YAG to alter their individual emission spectra. Further work on KSF/YAG composite phosphor ceramics is required to demonstrate that it is a viable alternative to phosphor in silicone, PiG, single-constituent ceramics, or single crystals, which will include integration into a prototype device, measurement of the quantum efficiency, and assessment of the performance as a function of light flux and temperature.

## 5. Patents

Systems and methods for fluoride ceramic phosphors for LED lighting, N.J. Cherepy, R.A. Osborne, S.A. Payne, Z. Seeley, A.M. Srivastava, W.W. Beers, W.E. Cohen (Lawrence Livermore National Security, LLC, Current Lighting Solutions, LLC), U.S. 11862758-B2, 2024.

## Figures and Tables

**Table 1:** Parameters used to model light emitted by YAG/KSF composite irradiated with a blue diode.

**KSF Parameters [21]**	
KSF emission wavelength, λ_KSF_	625 nm
KSF absorption cross-section, σ_Mn_ at 450 nm	1.50 × 10^−19^ cm^2^
KSF refractive index (589 nm)	1.34
KSF density, ρ_KSF_	2.75 g/cm^3^
KSiF_6_ molecular weight	220 g/mol
Mn atomic mass, m_Mn_	54.9 g/mol
**YAG parameters [23]**	
YAG emission wavelength (nm)	550 nm
YAG absorption cross-section, σ_Ce_ at 450 nm	3.0 × 10^−18^ cm^2^
YAG refractive index (589 nm)	1.83
YAG density, ρ_YAG_	4.56 g/cm^3^
Y_3_Al_5_O_12_ molecular weight	594 g/mol
Ce atomic mass, m_Ce_	140.1 g/mol

**Table 2:** R^2^ values for the model emission spectra versus the experimental data.

Thickness	R-Squared			
[mm]	2.5 wt% YAG	4.0 wt% YAG	5.0 wt% YAG	7.5 wt% YAG
1.7	0.88	0.95	0.95	0.91
1.0	0.77	0.94	0.95	0.97
0.7	0.79	0.90	0.95	0.97

**Table 3:** Color temperature, CRI, R9, efficacy, and CIE color coordinates of the samples.

YAG Loading	Thickness	CCT (K)	CRI	R9	Efficacy (lm/W)	CIE Coordinates	
						x	y
2.5 wt% YAG	1.7 mm	2716	92.6	77.6	128.2	0.452	0.399
	1 mm	3357	91.7	80.3	114.4	0.388	0.331
	0.7 mm	5380	75.6	−6	99.7	0.335	0.269
4 wt% YAG	1.7 mm	2646	76.2	22	146.7	0.499	0.473
	1 mm	2854	79.9	27.9	145	0.473	0.458
	0.7 mm	3258	84.6	45.6	134.5	0.421	0.401
5 wt% YAG	1.7 mm	2594	74.2	20.6	146.6	0.504	0.474
	1 mm	2724	76.7	22.2	147.5	0.492	0.474
	0.7 mm	3004	79.6	23.5	145.9	0.46	0.454
7.5 wt% YAG	1.7 mm	2450	69.4	−3	144.3	0.515	0.469
	1 mm	2726	69.6	−7.4	150.3	0.495	0.479
	0.7 mm	2927	73.4	−3.9	150.2	0.474	0.47

## Data Availability

All data are contained in the article.
